# Aldose Reductase Inhibitory Activity of Compounds from  *Zea mays* L.

**DOI:** 10.1155/2013/727143

**Published:** 2013-03-17

**Authors:** Tae Hyeon Kim, Jin Kyu Kim, Young-Hee Kang, Jae-Yong Lee, Il Jun Kang, Soon Sung Lim

**Affiliations:** ^1^Department of Food Science and Nutrition, Hallym University, Chuncheon 200-702, Republic of Korea; ^2^Institute of Natural Medicine, Hallym University, Chuncheon 200-702, Republic of Korea; ^3^Department of Biochemistry, College of Medicine, Hallym University, Chuncheon 200-702, Republic of Korea

## Abstract

Aldose reductase (AR) inhibitors have a considerable therapeutic potential against diabetes complications and do not increase the risk of hypoglycemia. Through bioassay-guided fractionation of an EtOH extract of the kernel from purple corn (*Zea mays* L.), 7 nonanthocyanin phenolic compounds (compound **1–7**) and 5 anthocyanins (compound **8–12**) were isolated. These compounds were investigated by rat lens aldose reductase (RLAR) inhibitory assays. Kinetic analyses of recombinant human aldose reductase (rhAR) were performed, and intracellular galactitol levels were measured. Hirsutrin, one of 12 isolated compounds, showed the most potent RLAR inhibitory activity (IC_50_, 4.78 **μ**M). In the kinetic analyses using Lineweaver-Burk plots of 1/velocity and 1/substrate concentration, hirsutrin showed competitive inhibition against rhAR. Furthermore, hirsutrin inhibited galactitol formation in rat lens and erythrocytes sample incubated with a high concentration of galactose; this finding indicates that hirsutrin may effectively prevent osmotic stress in hyperglycemia. Therefore, hirsutrin derived from *Zea mays* L. may be a potential therapeutic agent against diabetes complications.

## 1. Introduction

Trials on diabetes control and complications show that hyperglycemia is a key factor in the development of diabetic complications. Diabetic complications occur in many tissues and affect various sensory organs, the nervous system, circulation, and renal excretion [1–3].

Diabetic complications can arise from increased flux through the polyol pathway. Aldose reductase (AR) (alditol: NAD (P)^+^ 1-oxidoreductase, EC 1.1.1.21) is a reduced form of nicotinamide adenine dinucleotide phosphate (NADPH) specific aldo-keto oxidoreductase used to catalyze the conversion of glucose to galactitol in the polyol pathway, which is the alternate route for the metabolism of a small amount of nonphosphorylated glucose [4]. Because polyols such as galactitol do not readily move across the cell membranes, they can cause severe osmotic stress, leading to cell swelling and damage.

 This pathway plays an important role in the etiology of complications of diabetes, such as neuropathy, cataract formation, nephropathy, and retinopathy. Therefore, inhibitors of AR may have the potential to treat long-term diabetic complications [5]. 

Although the development and progression of diabetic complications can be prevented by controlling blood glucose, it is difficult to regulate normal blood glucose in a diabetic patient. Therefore, AR inhibition has been recognized as an important strategy in the prevention and attenuation of long-term diabetic complications; further, AR inhibitors are being studied as potential therapeutics against diabetic complications [5, 6].

Purple corn (*Zea mays *L.) has been cultivated for centuries in the Andean region. Peruvian people consume a typical drink made from purple corn called “chicha morada,” a folk medicine that is believed to improve health. Purple corn is an important source of anthocyanins, which have potential applications as natural food colorants and antioxidants [7]. The flavonoid pigments present in a wide range of plant products are considered to have antioxidant effects that inhibit cell mutation and reduce chemically induced colorectal carcinogenesis, but they may also prevent diabetes [8–10]. 

Beneficial health-related effects of other nonanthocyanin phenolic compounds (dehydrodieugenol, trans-coniferyl aldehyde, trans-coniferyl alcohol, condensed tannin, catechin, *p*-hydroxybenzoic, protocatechuic, *p*-coumaric, syringic, caffeic, and ferulic acids) have also been extensively reported including antioxidant [11–13], antimutagenic [14, 15], anticarcinogenic activity [16–18], and other biological properties.

In general, corn is considered an important crop worldwide. There have been some studies in the identification and quantification of phenolic compounds from yellow corn including *p*-hydroxybenzoic, protocatechuic, *p*-coumaric, and ferulic acids [11–13]. However, the bioactivity of nonanthocyanin phenolic compounds present in purple corn has not been reported to our knowledge.

In this paper, we report the inhibition of rat lens aldose reductase (RLAR) by an ethanol extract of the kernel of *Zea mays *L. (purple corn), the isolation of active compounds, and their structure-activity relationships. We also investigated the effects of these compounds on galactitol accumulation in rat lens and erythrocytes.

## 2. Materials and Methods

### 2.1. Chemicals

^1^H- and ^13^C-NMR spectra were recorded with a Bruker Avance 600 MHz NMR spectrometer (karlsruhe, Germany) operating at 600 and 150 MHz, respectively. The first grade solvents were used for extraction, fractionation, and column chromatography. RP C-18 (40–75 mesh, Nacalai tesque, Japan) was used as the column packing material. DL-Glyceraldehyde, D-glucose, bovine serum albumin (BSA), nicotinamide adenine dinucleotide phosphate (NADPH), and quercetin were purchased from Sigma Chemical (St. Louis, MO, USA). Recombinant human aldose reductase (rhAR) was purchased from Wako Pure Chemical Industries (Osaka, Japan). All tested compounds were isolated from the kernel of purple corn (Session 2.3.), and the structures of the compounds were elucidated using mass spectrometry and NMR spectroscopy: protocatechuic acid (compound no. **1**, MF: C_7_H_6_O_4_, Mw: 154.12 g/mol, 98.6% in HPLC), vanillic acid (**2**, MF: C_8_H_8_O_4_, Mw: 168.15, 94.4%), 2,4,6-trihydroxybenzoic acid (**3**, MF: C_7_H_6_O_5_, Mw: 170.12, 97.8%), *p*-hydroxycinnamic acid (**4**, MF: C_9_H_8_O_3_, Mw: 164.16, 96.6%), ferulic acid (**5**, MF: C_10_H_10_O_4_, Mw: 194.18, 97.1%), hirsutrin (**6**, MF: C_21_H_20_O_12_, Mw: 464.10, 93.9%), 3′-methoxyhirsutrin (**7**, MF: C_22_H_22_O_13_, Mw: 494.10, 96.7%), cyanidin-3-glucoside (**8**, MF: C_21_H_22_O_11_, Mw: 448.41, 99.6%), pelargonidin-3-glucoside (**9**, MF: C_21_H_20_O_13_, Mw: 432.18, 98.4%), peonidin-3-glucoside (**10**, MF: C_22_H_24_O_11_, Mw: 464.15, 98.2%), cyanidin 3-(6′′-malonylglucoside) (**11**, MF: C_24_H_23_O_14_, Mw: 535.43, 98.9%), and pelargonidin 3-(6′′-malonylglucoside) (**12**, MF: C_24_H_23_O_13_, Mw: 519.0, 96.2%).

### 2.2. Plant Materials

Purple corn extract in a powdered form was provided by Brilliant Project Int'l Co., Ltd. (Seoul, Korea). The powder was transferred into a glass open column (1.0 × 900 mm i.d.), filled with Diaion HP-20 resin (Mitsubishi, Japan), and eluted with water to remove sugar and impurities, followed by ethanol to obtain the purple corn polyphenol extracts (PCPEs). The obtained PCPEs solution was evaporated and freeze-dried, and it was maintained at −20°C for further experiments.

### 2.3. Isolation and Identification

For solvent fractionation, PCPE (60 g) was suspended in water and then partitioned sequentially with *n*-hexane (Hex), methylene chloride (MC), ethyl acetate (EtOAc), and *n*-butanol (*n-*BuOH), leaving a residual aqueous fraction (Aq). Each fraction was then evaporated *in vacuo* to yield the Hex (0.33 g, 0.55%), MC (0.65 g, 1.08%), EtOAc (5.57 g, 9.28%), *n*-BuOH (26.44 g, 44.07%), and Aq (22.98 g, 38.30%) fractions.

**Figure 1:**
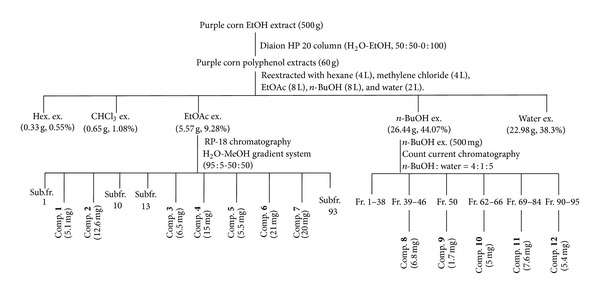
Isolation scheme for the compounds from purple corn (*Zea mays* L).

The EtOAc fraction (5.57 g) was subjected to C-18 gel (Lichroprep RP-18 (40–63 *μ*m); Merck, Darmstadt, Germany) column chromatography, eluted with water and increasing methanol concentration in an H_2_O-MeOH gradient system (95 : 5 → 50 : 50, v/v) to yield 93 subfractions as eluting solvents for the isolation of compounds **1** (5.1 mg), **2** (12.6 mg), **3** (6.5 mg), **4** (15.0 mg), **5** (5.5 mg), **6** (21.0 mg), and **7** (20.0 mg) (Figure 1). 

**Figure 2:**
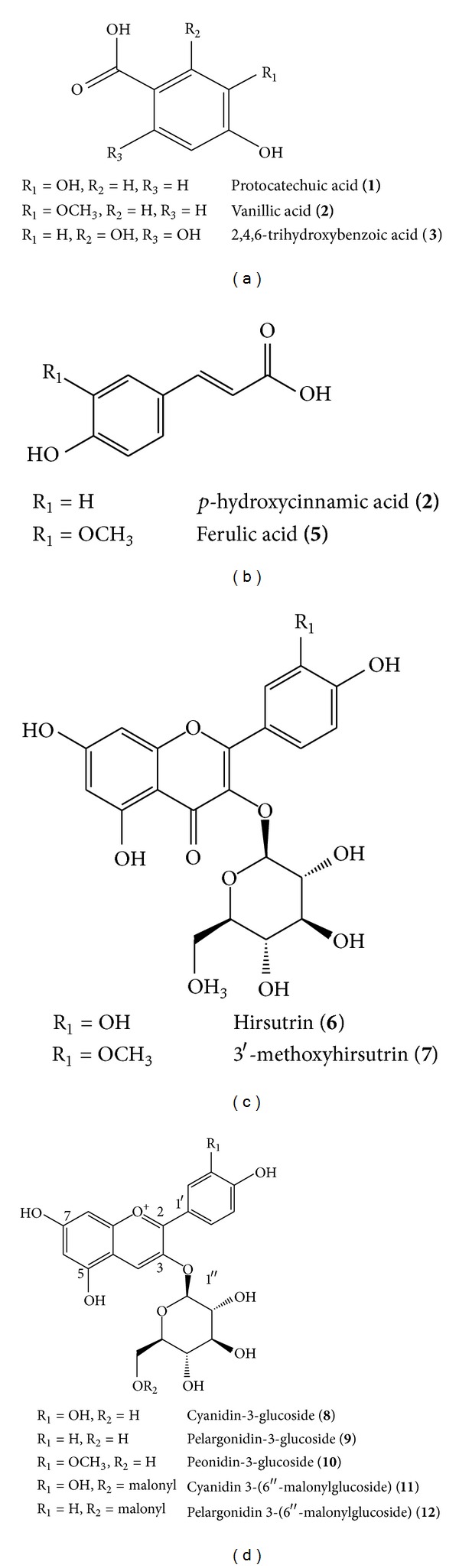
Chemical structures of phenolic compounds isolated from *Zea mays* L.

Anthocyanins were isolated following a modified procedure of that described by Renault et al. [19]. The *n*-BuOH fraction (500 mg) was subjected to high-speed countercurrent chromatography (HSCCC). The HSCCC system employed in the present study was a Model TBE-1000A HSCCC (Shanghai Tauto Biotechnique, Shanghai, China) with 3 multilayer coil columns connected in series and a 50-mL sample loop. The inner diameter of the PTFE tubing was 1.8 mm, and the total volume capacity was 1000 mL. The *b*-value of the preparative column varied from 0.42 at the internal layer to 0.63 at the external layer. The rotation speed of the apparatus was regulated, using a speed controller, to be in the range from 0 to 600 rpm. The HSCCC system was equipped with a Model Hitachi L-6200 intelligent pump (Hitachi, Tokyo, Japan), Model TOPAZ dual UV monitor operating at 520 nm, and Model ECOMAC-ECOM Acquisition and Control (version 0.97). The upper phase, consisting of a mixture of *n*-BuOH : acetic acid : water (4 : 1 : 5, v/v/v), was used as the stationary phase, while the lower phase was used as the mobile phase. The mobile phase had a flow rate of 2.5 mL/min; centrifugation was carried out at 400 rpm. Ten milliliters of each fraction was collected. Compounds **8** (6.8 mg), **9** (1.7 mg), **10** (1.7 mg), **11** (7.6 mg), and **12** (5.4 mg) were isolated (Figure 1) and identified by LC-MS/MS. These compounds were identified by comparing their NMR data with those in the literature and/or by directly comparing with their authentic samples. Their chemical structures are shown in Figure 2.

### 2.4. Measurement of RLAR Activity

RLAR activity was assayed according to the methods described by Dufrane et al. [20]. Rat lenses were removed from Sprague-Dawley rats weighting 250–280 g and frozen until required. The rat lens homogenate was prepared according to the method of Hayman and Kinoshita with some modifications [21–23]. A partially purified enzyme with a specific activity of 6.5 U/mg was routinely used to test the enzyme inhibitions. The partially purified material was separated into 1.0 mL aliquots and stored at −40°C. RLAR activity was assayed spectrophotometrically by measuring the decrease in absorption of NADPH at 340 nm over a 4-minute period with DL-glyceraldehyde as a substrate. Each 1.0 mL cuvette contained equal units of enzyme, 0.05 M sodium phosphate buffer (pH 6.2), and 0.3 mM NADPH with or without 10 mM substrate and inhibitor. For inhibition studies, concentrated stocks of AR inhibitors prepared in DMSO were used, and the final concentration of DMSO was not more than 1%. The concentration of inhibitors giving 50% inhibition of enzyme activity (IC_50_) was calculated from least-squares regression line of logarithmic concentrations plotted against the residual activity.

### 2.5. Kinetics of rhAR by Active Compounds

Reaction mixtures consisted of 0.1 M potassium phosphate, 0.16 mM NADPH, and 2 mM of rhAR with varied concentrations of substrate DL-glyceraldehyde and AR inhibitor in a total volume of 200 *μ*L. Concentrations were ranged from 0.1 to 1.0 mM for DL-glyceraldehyde. rhAR activity was assayed spectrophotometrically by measuring the decrease in absorption of NADPH at 340 nm after substrate addition using Biotek Power Wave XS spectrophotometer (Biotek instruments, VT, USA) [24]. *K*_*m*_ and *V*_max⁡_ of rhAR were determined with varying concentrations of DL-glyceraldehyde as a substrate in the absence and presence of different concentrations of inhibitor by Lineweaver-Burk double reciprocal plots. Inhibitory constant (*K*_*i*_) was derived by plotting slopes obtained from Lineweaver-Burk plots versus inhibitors concentration.

### 2.6. Lens Culture and Intracellular Galactitol Measurement

Lenses isolated from 10-week-old male rats were cultured for 6 d in TC-199 medium that contained 15% fetal bovine serum 100 U/mL penicillin and 0.1 mg/mL streptomycin under sterile conditions in an atmosphere of 5% CO_2_ and 95% air at 37°C. Samples were dissolved in DMSO. The lenses were divided into 3 groups and cultured in medium containing 5 mM glucose, 30 mM galactose and quercetin (positive control), hirsutrin, or 3′-methoxyhirsutrin. Each lens was placed in well containing 1.0 mL of medium. Lenses from each group were minced in 150 *μ*L of phosphate-buffered saline on ice, homogenized by sonication, and centrifuged at 22.000 g for 10 min at 4°C. The supernatant was filtered through a membrane filter (Ultrafree-MC, nominal molecular weight limit 10,000; Millipore, Bedford, MA, USA) to remove proteins [25]. HPLC analysis for sugar alcohol in lens was performed with this filtrate after being benzoylated [26].

### 2.7. Blood Culture and Intracellular Galactitol Measurement

Blood sample was collected in heparin-containing polypropylene tube from 10-week-old male rats. For sugar and sugar alcohol analysis, erythrocytes from heparinized blood were separated from the plasma and buffy coat by centrifuging at 2000 ×g for 10 min. The cells were washed thrice with normal saline (0.9% NaCl) at 4°C. In the final washing, the cells were centrifuged at 2000 ×g for 10 min to obtain a consistently packed cell preparation. The packed cells (1 mL) were then incubated in a Krebs-Ringer bicarbonate buffer (pH 7.4) containing 30 mM galactose in the presence or absence of samples at 37°C in 5% CO_2_ for 3 h. The erythrocytes were washed with cold saline by centrifuging at 2000 ×g for 10 min, precipitated by adding 6% of cold perchloric acid (3 mL), and centrifuged again at 2000 ×g for 10 min. The supernatant was neutralized with 2.5 M K_2_CO_3_ at 4°C and used for galactitol determination [27]. HPLC analysis for sugar and sugar alcohol in blood was performed with this supernatant of red blood cell homogenate after being benzoylated [25].

## 3. Results and Discussion

The current study aimed to identify potential AR inhibitors from *Zea mays *L. that would be useful in the treatment of diabetic complications. To identify the active compounds from *Zea mays *L., the extract was divided into 5 systematic fractions, which were then tested for RLAR inhibitory activity using DL-glyceraldehyde as a substrate (Table 1). Of these fractions, the ethyl acetate (EtOAc) fraction was found to exhibit the strongest RLAR inhibitory activity with a mean IC_50_ value of 2.06 *μ*g/mL, whereas the positive control quercetin showed an IC_50_ value of 2.34 *μ*g/mL. This suggested the presence of ARIs in the EtOAc soluble fraction, so we focused on isolating ARIs from this fraction.

We isolated pure compounds by subjecting the EtOAc fraction to repeated chromatography on the RP C-18 chromatography. The chemical structures of 7 isolated compounds were identified. Chemical structures were elucidated by chemical and spectral analysis as protocatechuic acid (**1**), vanillic acid (**2**), 2,4,6-trihydroxybenzoic acid (**3**), 4-hydroxycinnamic acid (**4**), ferulic acid (**5**), hirsutrin (**6**), and 3′-methoxyhirsutrin (**7**). The *n*-BuOH fraction was subjected to HSCCC separation and yielded 5 compounds. Their chemical structures were identified by LC-MS as cyanidin-3-glucoside (**8**), pelargonidin-3-glucoside (**9**), peonidin-3-glucoside (**10**), cyanidin-3-(6′′-malonylglucoside) (**11**), and pelargonidin-3-(6′′-malonylglucoside) (**12**). The chemical structures of anthocyanins isolated are shown in Figure 2. 

The RLAR inhibitory activities of the 12 flavonoids isolated from the EtOAc and *n*-BuOH fractions of *Zea mays *L. are shown in Table 2. Of the compounds isolated from the *n*-BuOH fraction, cyanidin-3-(6′′-malonylglucoside) (**11**) showed RLAR inhibitory activity with an IC_50_ value of 11.74 *μ*M. Compounds **8**, **9**,** 10**, and **12** did not RLAR inhibitory activity. Hirsutrin (**6**) and 3′-methoxyhirsutrin (**7**) showed a good activity with IC_50_ values of 4.78 *μ*M and 5.67 *μ*M, respectively (the IC_50_ value of the positive control, quercetin, was 6.92 *μ*M).

Yawadio et al. isolated 2 anthocyanins (cyanidin-3-glucoside and peonidin-3-glucoside) and a phenolic compound (ferulic acid) from black and pigmented brown rice, and their complete structures were determined by spectroscopic analysis (NMR, and MALDI-TOF MS) [28]. The aldose reductase inhibitory activity of isolated compounds from black and pigmented brown rices was in the following decreasing order: cyanidin-3-glucoside > quercetin > ferulic acid > peonidin-3-glucoside. 

In the previous study, we postulated the following relationships of structure to the inhibitory activities of flavonoids: (1) the flavones and flavonols having the 7-hydroxyl; (2) flavones and flavonols with a catechol moiety at the B ring (the 3′,4′-dihydroxyl moiety) show stronger activity; (3) the 5-hydroxyl moiety does not affect the activity; (4) the 2-3-double bond enhances the activity than other flavonoids [9, 29–31].

**Figure 3:**
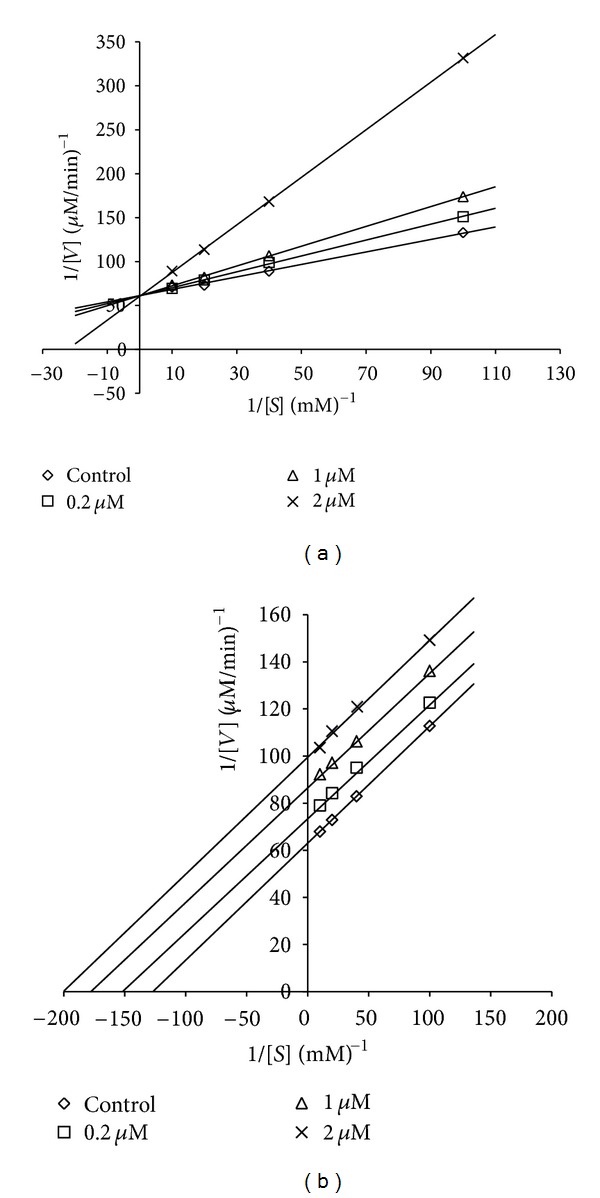
Lineweaver-Burk plots showing the reciprocal of the velocity (1/*V*) of recombinant human aldose reductase versus the reciprocal of substrate concentration (1/*S*) with dL-glyceraldehyde as the substrate concentration from 0.1 to 1 mM hirsutrin (a), 3′-methoxyhirsutrin (b).

In order to determine the type of inhibition activity on rhAR for compounds** 6** and **7**, a kinetic study was conducted using DL-glyceraldehyde as the substrate (concentration, 0.1–1.0 mM), with 3 different concentrations for each compound. The Lineweaver-Burk plots (1/velocity and 1/concentration) for compounds **6** and **7** are shown in Figure 3. As the concentration of the inhibitor compound **6** increases, steeper and steeper lines are obtained in a double-reciprocal plot. These lines were all intersecting at the same point on the *y*-axis, 1/*V*_max⁡_. The maximum velocity (*V*_max⁡_) of the reaction is unchanged, while *K*_*m*_ (Michaelis-Menten constant) is decreased. The result indicated that the inhibition type of rhAR by compound **6** was competitive. Further, we have determined inhibitory constant (*K*_*i*_) from secondary plots of Lineweaver-Burk plots, and *K*_*i*_ of compound **6** for rhAR was found to be 7.21 × 10^−7^ M. In case of compound **7**, the slopes were found to be parallel. *V*_max⁡_ and *K*_*m*_ were decreased with glyceraldehydes as substrates (Figure 3). The result indicated that the inhibition type of rhAR by compound **7** was uncompetitive, that is, this inhibitor could bind neither to the substrate-binding region nor to the NADPH-binding region of rhAR. As reported by Bohren and Grimshaw [32], although many ionic inhibitors bind to active site, still they show noncompetitive to uncompetitive pattern inhibition because under steady-state conditions most of the enzyme will be present as enzyme-nucleotide binary complex. Hence, compounds that selectively bind to the enzyme-nucleotide complex are more effective than those bind to free enzyme.

We also investigated the effect of flavonoids (**6** and **7**) isolated from *Zea mays *L. on the accumulation of galactitol in rat lens and erythrocytes. The primary cause of complications of diabetes is an elevated level of plasma glucose. The excess glucose causes flux through the polyol pathway, which results in a marked increase in AR activity and the accumulation of sorbitol, the polyol of glucose, in all tissues that do not require insulin for glucose uptake. There is a greater accumulation of polyol by the reduction of galactose than glucose owing to the higher affinity of aldose reductase for galactose than for glucose and the fact that there is no subsequent metabolism of galactitol [33]. The effects of compounds **6** and **7** on galactitol accumulation in rat erythrocytes are shown in Table 3. Galactitol accumulation was 22-fold greater when cells were incubated in a high-galactose medium, compared with incubation in a galactose-free medium. Compounds **6** and **7** effectively inhibited galactitol accumulation in rat erythrocyte by almost 32.54% and 11.96% at 50 *μ*g/mL, respectively. The positive control, quercetin, inhibited galactitol accumulation in rat erythrocytes by 30.48%, whereas a high-galactose culture medium reduced the galactitol level. The effects of compounds **6** and **7** on galactitol accumulation in rat lens are also shown in Table 3. Compound **6** (hirsutrin) effectively inhibited galactitol accumulation in rat lens by almost 33.78% at 5 *μ*g/mL. Quercetin, the positive control, inhibited by 46.82%.

Finally, the study of the selectivity of isolated compounds on aldehyde reductase could help to elucidate whether or not they have some deleterious side effects on human body.

## 4. Conclusion

In this study, we discovered that phenolic compounds isolated from *Zea mays *L. inhibit RLAR. We also found that phenolic compounds significantly suppressed galactitol accumulation in rat lens and erythrocytes. These results indicated that RLAR inhibitors isolated from *Zea mays *L. are effective in either preventing or retarding complications associated with diabetes.

## Figures and Tables

**Table 1:** Inhibitory effects of the kernel of *Zea mays *L. extracts on rat lens aldose reductase (RLAR).

Extracts and fractions	Concentration (*μ*g/mL)	Inhibition (%)	IC_50_ (*μ*g/mL)
Purple corn EtOH ext.	10	62.46	
	5	39.0	6.72
	1	3.18	
Purple corn polyphenol extracts (PCPEs)	10	78.53	
	5	58.18	3.72
	1	12.37	
PCPE—hexane fr.	10	66.13	
	5	39.13	7.28
	1	5.85	
PCPE—methylene chloride fr.	10	71.09	
	5	53.85	4.30
	1	13.38	
PCPE—ethyl acetate fr.	5	71.91	
	1	32.88	2.06
	0.5	13.84	
PCPE—*n*-butanol fr.	10	30.19	>10
PCPE—water fr.	10	20.61	>10
Quercetin^a^	10	82.99	
	5	67.91	2.34
	1	30.34	

Inhibition rate was calculated as percentage with respect to the control value and expressed as mean of triplicate experiments. The concentration of each test sample giving rise to 50% inhibition of activity (IC_50_) was estimated from the least-squares regression line of the logarithmic concentration plotted against inhibitory activity. ^a^Quercetin was used as positive control.

**Table 2:** Inhibitory effects of compounds isolated from the kernel of *Zea mays *L. on rat lens aldose reductase (RLAR).

Compounds	Concentration (*μ*g/mL)	Inhibition (%)	IC_50_	
			(*μ*g/mL)	(*μ*M)
Protochatechuic acid (**1**)	10	3.22	>10	
Vanillic acid (**2**)	10	20.34	>10	
2,4,6-trihydroxybenzoic acid (**3**)	10	73.73		
	5	48.14	5.92	34.82
	1	16.95		
*p*-hydroxycinnamic acid (**4**)	10	77.81		
	5	43.73	6.02	36.71
	1	13.76		
Ferulic acid (**5**)	10	77.25		
	5	49.83	4.24	21.85
	1	11.15		
Hirsutrin (**6**)	10	86.69		
	5	76.15	2.22	4.78
	1	27.56		
3′-methoxyhirsutrin **(7)**	10	89.74		
	5	73.67	2.71	5.67
	1	16.43		
Cyanidin-3-glucoside (**8**)	40	47.72	>40	
Pelargonidin-3-glucoside (**9**)	40	13.21	>40	
Peonidin-3-glucoside (**10**)	40	36.98	>40	
Cyanidin-3-(6′′-malonylglucoside) (**11**)	10	67.96		
	5	47.78	6.27	11.74
	1	19.01		
Pelargonidin-3-(6′′-malonylglucoside) (**12**)	10	NA^b^	>10	
Quercetin^a ^	10	82.99		
	5	67.91	2.34	6.92
	1	30.34		

Inhibition rate was calculated as percentage with respect to the control value and expressed as mean of triplicate experiments. The concentration of each test sample giving rise to 50% inhibition of activity (IC_50_) was estimated from the least-squares regression line of the logarithmic concentration plotted against inhibitory activity. ^a^Quercetin was used as positive control. ^b^NA means nonactive.

**Table 3:** Inhibitory effects of the compounds on the galactitol accumulation in rat lenses and erythrocytes.

Compounds	Galactitol content (*μ*M)^a^ in rat erythrocyte [inhibition %]	Galactitol content *μ*g/lens wet weight (g)^b^ [inhibition %]
Galactose free	0.98	—
Control	22.80	844.05 ± 74.02
Quercetin^c^	16.15 [30.48%]	448.81 ± 69.43 [46.82%]
Hirsutrin (**6**)	15.70 [32.54%]	558.92 ± 89.12 [33.78%]
3′-methoxyhirsutrin **(7)**	20.19 [11.96%]	828.35 ± 73.27 [1.86%]

^a^Erythrocyte was incubated in a Krebs-Ringer bicarbonate buffer containing 30 mM galactose and in the presence or absence of 50 *μ*g/mL compounds. ^b^Mean ± standard deviation of triplication analysis of rat lens with compounds at a concentration 5 *μ*g/mL. ^c^Quercetin were used as positive control.
